# Human Neural Stem Cell Extracellular Vesicles Improve Tissue and Functional Recovery in the Murine Thromboembolic Stroke Model

**DOI:** 10.1007/s12975-017-0599-2

**Published:** 2017-12-28

**Authors:** Robin L. Webb, Erin E. Kaiser, Shelley L. Scoville, Tyler A. Thompson, Sumbul Fatima, Chirayukumar Pandya, Karishma Sriram, Raymond L. Swetenburg, Kumar Vaibhav, Ali S. Arbab, Babak Baban, Krishnan M. Dhandapani, David C. Hess, M. N. Hoda, Steven L. Stice

**Affiliations:** 1 ArunA Biomedical, Athens, GA 30602 USA; 2 Regenerative Bioscience Center, Rhodes Center for Animal and Dairy Science, University of Georgia, Athens, GA 30602 USA; 3 Department of Medical Laboratory, Imaging, and Radiologic Sciences, Augusta University, Augusta, GA 30912 USA; 4 Cancer Center, Augusta University, Augusta, GA 30912 USA; 5 Department of Oral Biology, Dental College of Georgia, Augusta University, Augusta, GA 30912 USA; 6 Department of Neurosurgery, Augusta University, Augusta, GA 30912 USA; 7 Department of Neurology, Augusta University, Augusta, GA 30912 USA

**Keywords:** Neural stem cell extracellular vesicles, Thromboembolic stroke, Preclinical stroke model

## Abstract

Over 700 drugs have failed in stroke clinical trials, an unprecedented rate thought to be attributed in part to limited and isolated testing often solely in “young” rodent models and focusing on a single secondary injury mechanism. Here, extracellular vesicles (EVs), nanometer-sized cell signaling particles, were tested in a mouse thromboembolic (TE) stroke model. Neural stem cell (NSC) and mesenchymal stem cell (MSC) EVs derived from the same pluripotent stem cell (PSC) line were evaluated for changes in infarct volume as well as sensorimotor function. NSC EVs improved cellular, tissue, and functional outcomes in middle-aged rodents, whereas MSC EVs were less effective. Acute differences in lesion volume following NSC EV treatment were corroborated by MRI in 18-month-old aged rodents. NSC EV treatment has a positive effect on motor function in the aged rodent as indicated by beam walk, instances of foot faults, and strength evaluated by hanging wire test. Increased time with a novel object also indicated that NSC EVs improved episodic memory formation in the rodent. The therapeutic effect of NSC EVs appears to be mediated by altering the systemic immune response. These data strongly support further preclinical development of a NSC EV-based stroke therapy and warrant their testing in combination with FDA-approved stroke therapies.

**Electronic supplementary material:**

The online version of this article (10.1007/s12975-017-0599-2) contains supplementary material, which is available to authorized users.

## Introduction

Despite the overwhelming global need, intravenous tissue plasminogen activator (IV-tPA) and endovascular thrombectomy (ET) are the only two FDA-approved stroke therapies to date [1, 2]. Both of the above “reperfusion” therapies target opening of major blood vessels in a carefully diagnosed, yet a very small sub-population of stroke victims. While reperfusion could itself trigger a secondary injury, neither of the FDA-approved stroke therapies are directly neuroprotective or neuroregenerative. Moreover, the use of IV-tPA and/or ET is improbable as a field therapy and both are limited to state-of-the-art facilities [3, 4]. Therefore, a larger population of stroke patients with limited access to these facilities (e.g., rural populations) still remain untreated and often rely on later neurorehabilitation and endogenous neuroregeneration mechanisms [5, 6].

Ideally, an implementable therapy would protect the brain in acute stroke and enhance long-term functional outcomes among stroke survivors. Along these lines, the Stroke Treatment Academic Industry Roundtable (STAIR) recommends development of stroke therapies, which could reduce reperfusion injury and promote neurovascular plasticity and recovery later. An assessment of the litany of failed treatments by the Stem Cell Emerging Paradigm in Stroke Consortium meetings (STEPS I, II, and III) resulted in identifying major treatment deficiencies including (1) lack of a regenerative therapy that will not only protect cells from ischemic injury but stimulate regeneration of lost and damaged tissues and (2) translational animal models more reflective of human pathology and improved predictive testing of treatments [7, 8].

One of the most promising therapeutic avenues capable of addressing this need for a neuroprotective and/or regenerative therapy is the use of extracellular vesicles (EVs) [9]. EVs are membrane shed microvesicles (50–1000 nm) and exosomes (40–150 nm) produced by all cells of the central nervous system (CNS) [10, 11]. The therapeutic development of EVs is being explored for multiple regenerative therapeutic scenarios, as EVs overcome many of the limitations of cell therapies, including but not limited to the ability to deliver multiple doses, as well as the ability to store and administer EVs without specialized equipment or advanced training for medical personnel [12].

While reports on EV therapeutic benefits in rodent studies of mechanically occluded stroke (both transient suture and permanent electrocauterization models) are encouraging, optimal therapeutic EV sources have not been explored [13, 14]. Previously published stroke studies utilized non-neural sourced mesenchymal stem cell (MSC) EVs administered systemically into rodent models and produced behavioral improvements without significant reductions in infarct volume [13–15]. However, there are many indications that EV cargoes are cell type specific and the parental cell line plays a prodigious role in the biological properties of the resultant EV [14]. Therefore, EVs derived from different sources (MSC vs. NSC cells) may have unique properties relative to cell type. Also, the context under which EVs are produced directly influences the signal that the resultant EVs communicate [16, 17]. For example, EVs extracted from sera of stroke patients induced inflammatory cytokine expression in vitro [18]. Together, cell-specific activity and systemic immunological activation are novel multifaceted means by which EVs may provide beneficial effects in both local and systemic processes post-ischemic insult [19]. While specific mechanism(s) of action are still being investigated, the potential therapeutic mechanisms of EVs appear to include anti-oxidative, pro-angiogenic, immunomodulatory, and/or neural plasticity regulating processes [20, 21]. Additionally, since the majority of stroke (~ 87%) occurs due to a thromboembolic (TE) occlusion and a larger population of victims remains untreated with the FDA-approved reperfusion therapies, it is critical to validate this promising therapy in a physiologically relevant TE model of stroke [9, 22, 23].

The objective of this study was to evaluate the therapeutic potential of human neural stem cell-derived EVs in a highly relevant preclinical stroke model without immunosuppression. NSC EV treatment significantly decreased neural injury in the murine model of TE stroke and also resulted in decreased behavioral and motor function deficits.

## Results

### Pluripotent Stem Cell-Derived NSC and MSC EVs Were Similar in Structural and Protein Marker Expression But Not in Size

**Fig. 1:**
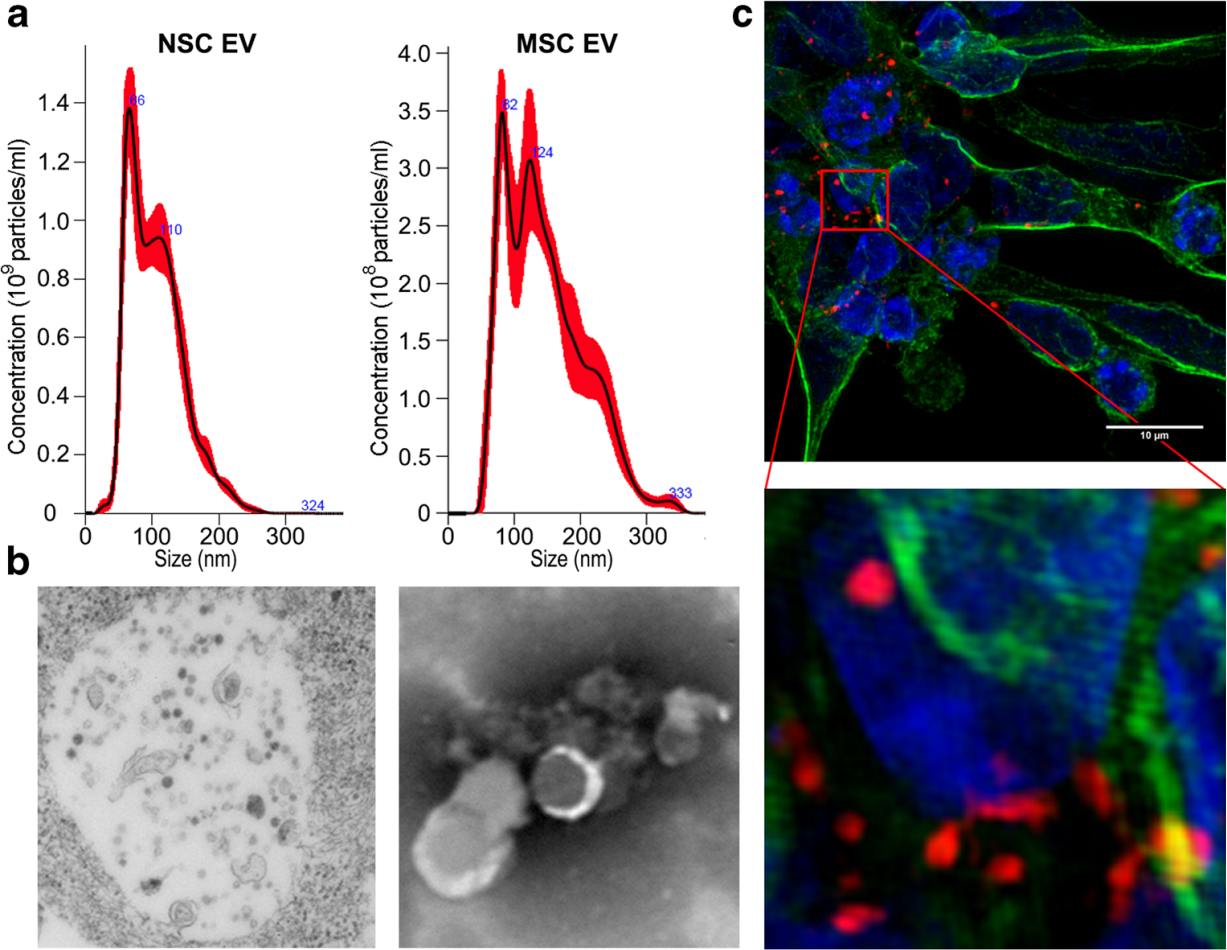
NSCs and MSCs produce EVs containing commonly reported EV biomarkers. EVs produced by NSCs and MSCs have overlapping, but distinct size profiles, with a greater presence of larger vesicles present in the MSC EV profile (~ 124 nm (**a**). Electron microscopy of NSC revealed the presence of disperse multivesicular bodies (**b**, left panel), while EV enrichment resulted in presence of purified EVs (**b**, right panel). Labeled NSC EVs (DiI) were taken up by differentiated neural cells in vitro (**c**), with single projection super resolution confocal images shown; z-stack in supplemental movie S1

To eliminate the potential confounding variable of genetic differences, NSC and MSC were isogenically derived from H9 pluripotent stem cells using processes previously developed [24–26]. NSC and MSC EVs were quantified and evaluated for size differences using Nanosight’s nanoparticle tracking analysis. NSC and MSC EVs have overlapping, but distinct size and concentration profiles, with a broader peak present in the MSC EV profile indicating presence of a range of vesicles up to 300 nm in size, while the vast majority of NSC EVs were under 200 nm (Fig. 1a). Evaluation of NSC EVs by electron microscopy (EM) revealed the presence of disperse multivesicular bodies (MVBs; Fig. 1b, left panel) and purified vesicles (Fig. 1b, right panel) could be visualized by EM after transfer to the electron microscopy grid. Differentiated neural cells were cultured with NSC EVs labeled with DiI and EVs were taken up by the neural cells in vitro, as shown in super resolution confocal microscopy projection images (Fig. 1c and enlarged inset). Analysis of EVs by flow cytometry revealed that both cell types produced EVs that contained similar amounts of commonly reported EV markers such as CD63 and CD81, which are both members of the highly conserved tetraspanin superfamily.

### NSC EVs Provided Significant Benefits in the Murine Embolic Model

**Fig. 2:**
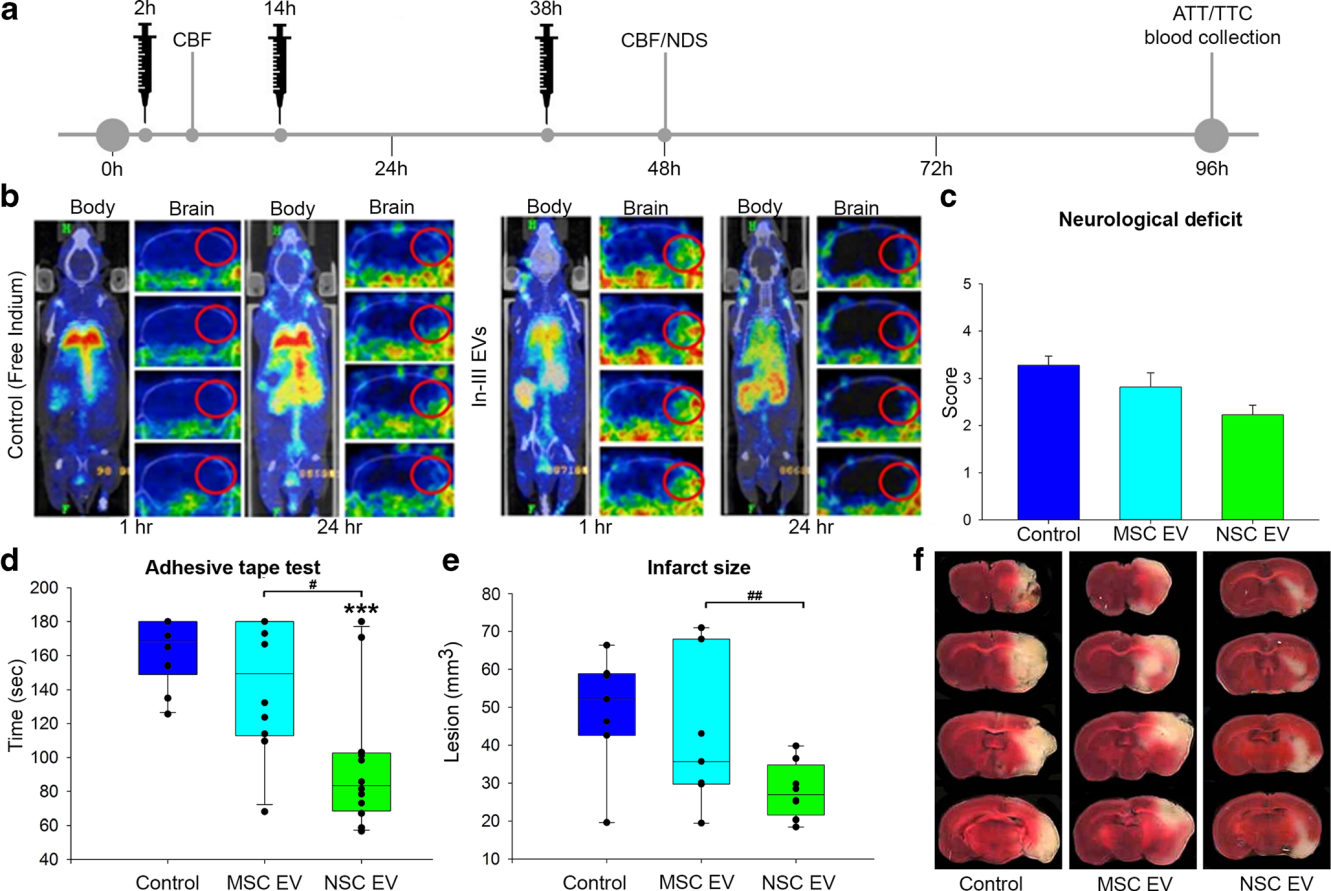
NSC EVs outperform MSC EVs in the murine embolic stroke model and indicate acute benefits may be modulated by augmenting the systemic immune response. One hour after stroke induction either free In-111 or labeled EVs (**b**, left and right, respectively) were administered into mice via tail vein injection and analyzed by SPECT. EVs were present in the infarct region 1 h after injection (**b**, red circles, left brain panels), but were largely cleared by 24 h (**b**, red circles, right brain panels). Systemic presence in the lungs, liver, and spleen are in agreement with other EV biodistribution studies (**b**, body panels). Based on rapid clearance, animals received three doses of EVs (MSC EV, NSC EV, or vehicle control; *N* = 12/group), at 2, 14, and 28 h after TE-MCAO, (as outlined in **a**). Neurological deficit 48 h post-TE-MCAO (**c**) indicated that animals that received MSC EVs were indistinguishable from controls, while NSC EV evaluation trended toward significance (*p* = 0.055). Adhesive tape test indicated improved somatosensory function after NSC EV treatment compared to either MSC EV or control (**d**) Acute effects on neural tissue were analyzed by 2,3,5-triphenyltetrazolium Chloride (TTC) differentiated metabolically active (live, red) and inactive (dead, colorless) tissue indicated significantly decreased injury and infarct following NSC EV treatment (**e**, **f**)

**Fig. 3:**
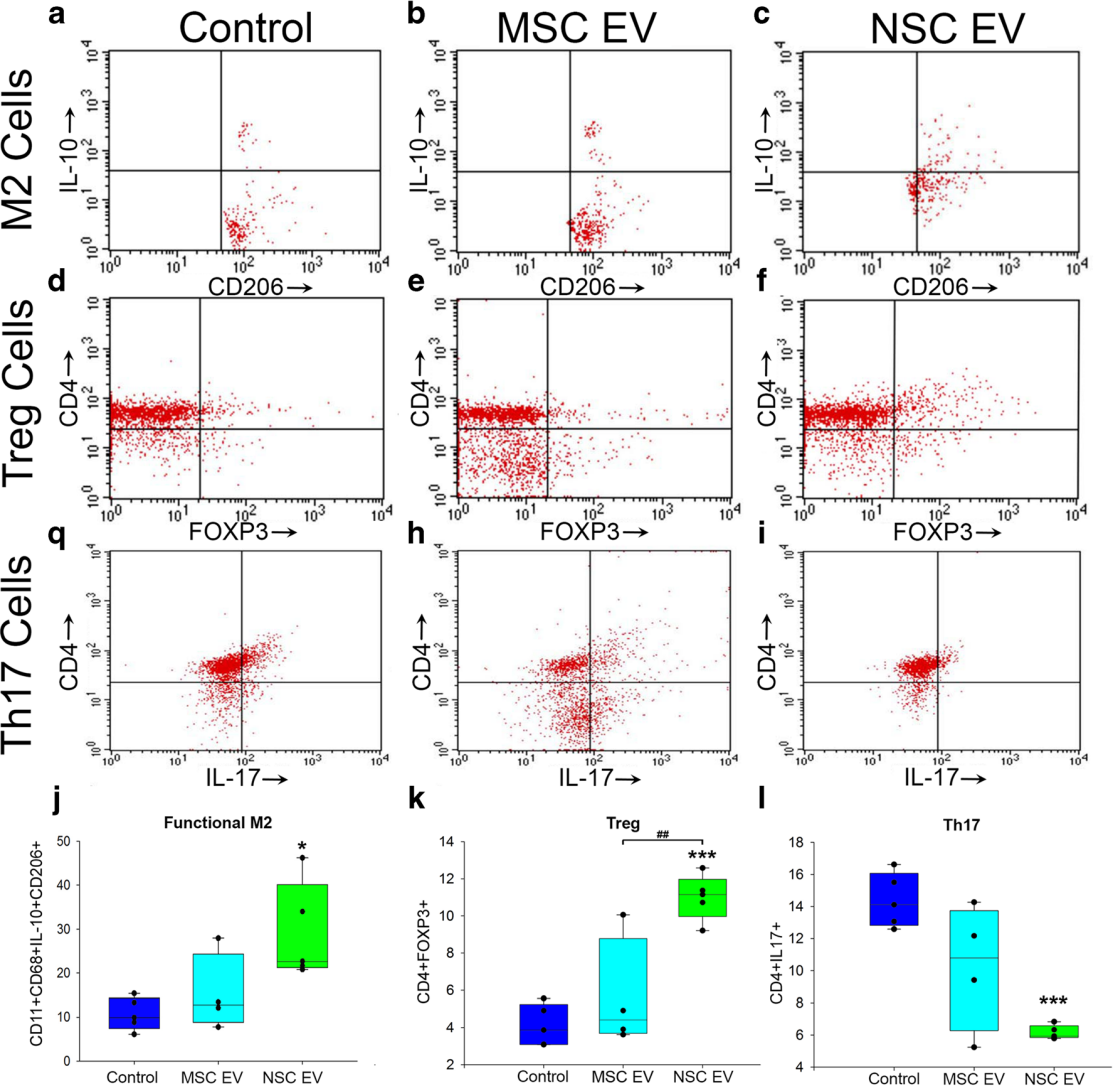
NSC EV treatment augments the systemic immune response to TE stroke. Cells in circulation that were analyzed for immune cell presence indicated an increase in functional M2 macrophages associated with tissue repair (**a**–**c**, **j**) and increased immunosuppressive Tregs (**d**–**f**, **k**), as well as a decrease in pro-inflammatory Th17 (CD4+, IL-17+) cells compared to MSC EVs and control (**g**–**i**, **l**). Asterisks (*) indicate statistical differences from sham group while the number sign (#) indicates significant statistical differences between control and NSC EV groups; *^;#^ *p* value ≤ 0.05; **^,##^ *p* value ≤ 0.01; ***^,###^ *p* value ≤ 0.001

In order to compare the therapeutic efficacy of isogenically derived NSC and MSC EVs side by side, EV biodistribution was first evaluated. Indium-111 (In-111)-labeled EVs were injected 1 h post-TE-MCAO. Animals were imaged by single photon emission computed tomography (SPECT) at 1 and 24 h post-injection (Fig. 2b) [27]. SPECT results demonstrated systemic distribution not only in the lungs, liver, and spleen, as reported in other EV biodistribution studies [16, 28], but were also present in the infarcted hemisphere by 1 h post-TE-MCAO. By 24 h, EVs were largely cleared from the infarct site, although still present in the other organs. These results suggest that EVs preferentially accumulate in the penumbra of the injury. Based on this clearance from the infarct, animals received a three-dose treatment regimen of either EVs or PBS vehicle by tail vein injection at 2, 14, and 38 h post-TE-MCAO. Animals were evaluated (after confirming no difference in cerebral blood flow; Fig. S1, a) by neurological deficit score (NDS) at 48 h and adhesive tape test (ATT) at 96 h post-TE-MCAO followed by blood collection and tissue analysis (Fig. 2a). NSC EV-treated animals during NDS assessment demonstrated a decrease in deficits compared to controls as evaluated by lower scores (*p* ≤ 0.055) Fig. 2c). NSC EV-treated animals performed significantly (*p* ≤ 0.001) faster on ATT (96.17 ± 11.57 vs. 162.53 ± 6.3 s, respectively), indicating enhanced sensorimotor function, when compared to controls or MSC EV-treated animals (Fig. 2d). Analysis of metabolically active tissue by 2,3,5-triphenyltetrazolium chloride (TTC) staining versus dead tissue (colorless) indicated significantly decreased tissue loss in NSC EV-treated animals compared to the MSC EV treatment group (27.97 ± 2.78 vs. 48.19 ± 5.79 mm^2^, Fig. 2e, f). Since EVs are present in bodily fluids and they could affect the systemic immune response via both direct and indirect antigen presentation, we next checked the peripheral immune response after EV treatment. Quantitative flow cytometry analysis of freshly collected blood samples at 96 h post-stroke indicated that NSC EV treatment significantly promoted macrophage polarization toward an anti-inflammatory M2 phenotype (Fig. 3a–c, j) and increased the regulatory T cell (Fig. 3d–f, k) population resulting in the downregulation of pro-inflammatory effector Th17 cells (Fig. 3g–i, l). Thus, our data indicates that NSC EV treatment after stroke is capable of dampening injury responses while augmenting a reparative systemic immune response (Fig. 3). In summary, this data indicates PSC-derived NSC EVs provide molecular and behavioral benefits, while PSC-derived MSC EV treatment resulted in more variable results in both infarct size and behavioral outcome assessment indicating a clear NSC EV benefit in the middle-aged embolic model. While overall survival was not significantly different between the groups, 55% of animals in the MSC EV and PBS groups survived to the endpoint, while 65% of NSC EV-treated mice survived (Fig. S1). For these reasons, NSC EVs were further explored as a candidate treatment, while evaluation of MSC EVs was discontinued.

### NSC EV Treatment Reduced Lesion Volume and Improved Behavioral Outcomes in Aged Mice

**Fig. 4:**
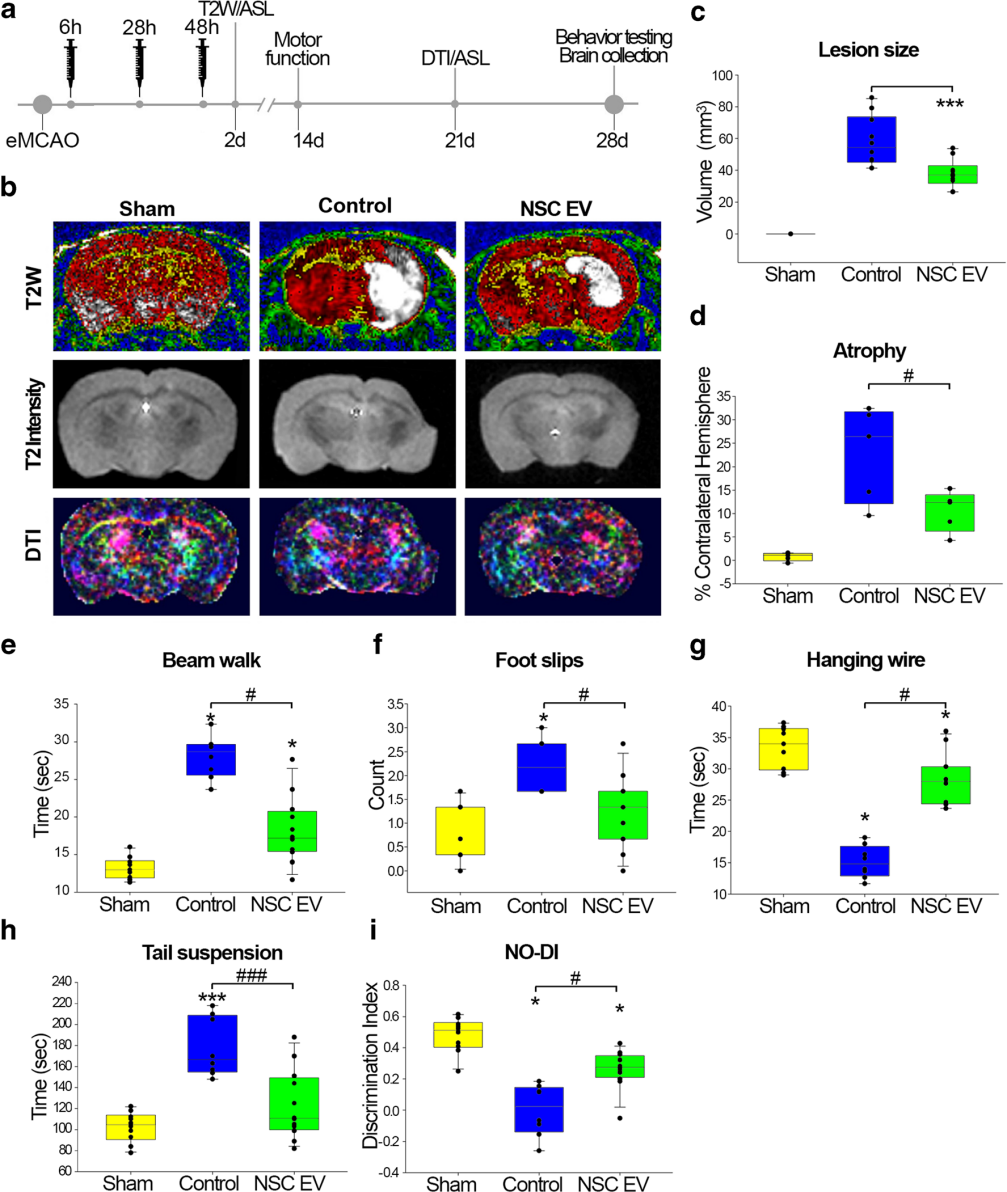
NSC EV treatment resulted in molecular and behavioral benefits in aged rodents. Based on increased benefit from NSC EV treatment, aged C57BL/6 animals (*N* = 24/group) were randomly split into control (PBS vehicle) and NSC EV treatment groups by blinded investigators, who delivered treatments at 6, 28, and 48 h as outlined in **a**. Analysis of T2 W images (**b**, lesion shown in white) demonstrated a significant reduction in lesion size in NSC EV-treated aged mice relative to control mice at 48 h (**c**). Volumetric analysis of T2 intensity (**b**) sequences revealed a significant reduction in ipsilateral hemisphere atrophy in NSC EV-treated mice relative to non-treated mice at 30 days (**d**). DTI sequences showed no significant differences in FA between NSC EV-treated and control mice at 28 days (**b**). Balance and coordination was evaluated by beam walk, where both TE-MCAO groups took longer to cross the beam than sham animals, but NSC EV-treated animals were significantly faster at performing the task than controls (**e**). The number of foot slips during beam walk also indicated improved coordination in treated animals vs. control (**f**). Forelimb coordination was further analyzed by hanging wire test, where NSC EV animals significantly outperformed control animals (**g**). Tail suspension test revealed that control animals were immobile for significantly longer than NSC EV-treated animals (**h**). Non-spatial memory of animals was evaluated by novel object recognition test, where NOR discriminatory index indicated that both TE-MCAO groups had detectable deficits, but NSC EV-treated mice performed significantly better than controls, as a result of treated animals spending more time with the novel object, compared to the familiar object (**i**). Asterisks (*) indicate statistical differences from sham group while the number sign (#) indicates significant statistical differences between control and NSC EV groups; *^;#^ *p* value ≤ 0.05; **^,##^ *p* value ≤ 0.01; ***^,###^ *p* value ≤ 0.001

Stroke therapeutics are often tested in young animals within a narrow time range post-stroke. NSC EVs were further explored in aged mice (18 ± 1 months), starting approximately 6 h post-stroke, to fall outside the time window of traditional tPA administration in humans. Dosage in the embolic model was maintained constant; however, the administration window was shifted to 6, 24, and 48 h post-stroke. (Fig. 4a). Blinded investigators randomly divided mice into non-stroked (sham) and stroked with either PBS vehicle (control) or NSC EV in PBS treatment groups (*N* = 24 animals/group). Analysis of T2-weighted (T2W) sequences 2 days post-TE-MCAO indicated a significant decrease in lesion volume in NSC EV-treated animals (58.2 ± 5.03 and 37.9 ± 2.84 mm^3^, respectively) (Fig. 4b, c), while ex vivo Q-ball MRI (performed on the fixed brain post-euthanasia) indicated that NSC EV treatment attenuated the post-stroke cerebral atrophy and significantly decreased it compared to the vehicle-treated group (22.8 ± 0.40 and 10.6 ± 1.94% of contralateral hemisphere) (Fig. 4d). Diffusion tensor imaging (DTI) and fractional anisotropy (FA) analysis was also performed after Q-ball imaging; however, no significant differences in diffusivity or white matter integrity were observed between the two groups subjected to TE stroke, which is likely due to less white matter content in small rodents.

Behavioral characteristics and motor function were evaluated 14 days post-TE-MCAO. NSC EV-treated animals exhibited significantly improved coordination on the balance beam relative to control, with NSC EV-treated animals crossing in 18.9 ± 1.36 s and control animals crossing in 28.0 ± 0.45 s (Fig. 4e). Significantly fewer foot slips while crossing the beam (2.21 ± 0.18 vs. 1.25 ± 0.21 foot slips) were also observed in NSC EV-treated animals (Fig. 4f). Grasping ability and forelimb strength were evaluated by the hanging wire test. NSC EV-treated animals could hang an average of 28.47 ± 1.18 s, while control animals grasping was significantly shorter (5.1 ± 0.91 s) (Fig. 4g). Episodic memory was evaluated by novel object recognition (NOR) testing. NSC EV-treated mice spent significantly more time exploring the novel object (NO; 36.92 ± 1.48 s) than the control group that spent only 26.50 ± 3.29 s on average with the NO. There were no significant differences in time spent with the familiar object between groups. Novel object discrimination index (DI) indicated NSC EV-treated animals performed significantly better than control group (0.26 ± 0.04 and 0.0005 ± 0.05, respectively; Fig. 4i). Finally, depressive phenotype was assessed by tail suspension test 28 days post-TE-MCAO. Controls were immobile for a significantly longer time period (178.13 ± 9.96 s) as compared to NSC EV-treated animals (123.08 ± 9.58 s) (Fig. 4h). The NSC EV group was not statistically different from the sham group in survival rates, while fewer animals survived to the endpoint in the control group (Fig. S1 a; *p* ≤ 0.319). Collectively, this data indicates an early neuroprotective effect of NSC EV in aged mice as indicated by reduced lesion volume and improvements in functional outcomes as measured by grasping ability, forelimb strength, motor coordination, and memory consolidation.

## Discussion

We present here the first experimental evidence that NSC EVs improve cellular, tissue, and functional outcomes in the murine TE-MCAO models. Mitigating the secondary injury cascades, particularly the immune response, NSC EV intervention led to significantly decreases in infarct size and brain atrophy, which has never been observed acutely in previous studies of exosome treatment for stroke [13–15]. Although various cell therapies have improved stroke recovery in preclinical models, NSC EVs possess a number of advantages over cell-based therapeutics including decreased tumorigenicity, limited immunogenicity, enhanced biodistribution, and BBB permeability [13, 29–31]. In addition, vesicles are involved in many biological processes with the potential to serve as a neuroprotective and translatable therapeutic for neural disabilities including ischemic stroke and, importantly, can likely be used in conjunction with currently available tPA and/or endovascular therapies [32, 33]. Tissue level changes generated large-scale reductions in neural injury and rapid recovery of neurological and motor function outcomes in vivo, thus suggesting NSC EVs are a promising therapeutic for human patients.

Functional benefits following MSC EV treatment for stroke has been evaluated using several different cell lines, with varying degrees of MSC marker definition and EV dose [13, 14, 34]. However, benefits in the infarct, including evidence of axonal remodeling and angiogenesis in the ischemic boundary zone were achieved using EVs from cells modified by a lentivirus, indicating that modification can influence therapeutic potential of the resultant EVs [34]. Uniquely, the MSC EVs tested were of PSC origin and differentiated in vivo. We have shown previously that although these cells have many of the common markers (CD73, CD93, and CD105), they can have unique differentiation potential and methylation patterns [35]. MSC sourced using different tissue origins, isolation methods, and in vitro culture conditions can alter the immunosuppression potency of MSC [36]. Thus, the results here may not represent results obtained by all sources of MSCs. However, these findings do elude to unknown subtleties of screening complex biologics, like EVs, for therapeutic potential in humans.

Stroke is unpredictable and the degree of neuroprotection provided by EVs may likely vary by the efficiency of their delivery into the ischemic brain. Therefore, we tested NSC EVs in two different treatment regimens in murine TE stroke. NSC EVs therapy, as early as 2 h after TE stroke in middle-aged (12 months old) mice, not only improved the neurological outcomes and profoundly reduced the infarction volume but also downregulated the systemic inflammatory response in the blood. It is well established that following stroke, immune cells such as leukocytes infiltrate the brain as a result of increased adhesion phenomena and resultant BBB permeability, leading to a brain localized neuroimmune response [37]. Circulating macrophages can also trigger a long-term adaptive immune response causing chronic neurodegeneration and subsequent neuropsychiatric dysfunction even after closure of the BBB [38]. Naïve immune cells such as macrophages and T lymphocytes are highly plastic in nature, which can adapt to a context-specific functional phenotype depending upon the microenvironment. Activated macrophages can also traverse into the draining cerebro-meningeal lymphatic system to trigger an adaptive immune response, which can decide the fate of outgoing T lymphocytes targeting the injured brain [39]. Since EVs carry a number of proteins, various RNA species, and bioactive lipids capable of diverse signaling, we looked into the systemic immune response 96 h after stroke. Mice treated with repeated doses of NSC EV showed increased M2-type macrophages and Treg populations, with a concurrent decrease in Th17 lymphocytes. Since macrophage activation precedes T lymphocyte proliferation and activation, it is likely that acute treatment with NSC EVs promoted a conducive microenvironment resulting in alternatively (but not classically) activated M2-type polarization. This likely skews T lymphocytes to their regulatory phenotype, (Treg) with concurrent suppression of pro-inflammatory Th17 (an effector phenotype which releases IL-17 and causes long-term neurodegeneration after stroke) [40]. Although these mechanistically novel findings in response to NSC EV therapy need further investigation, it is probable that such responses could have translational importance (Fig. 2); as such, circulating immune cells from the blood could possibly be used as a convenient biomarker to follow chronic effects of disease progression and the therapeutic effect of NSC EV in stroke during long-term follow-up.

Chronic neuropsychiatric dysfunctions such as the exacerbation of depression, anxiety, and dementia in aged individuals are very common after stroke [41]. Therefore, we next evaluated the delayed NSC EV therapy in the reproductively senescent aged (18 months old) mice subjected to TE stroke model and followed them for both acute and chronic outcomes. NSC EV therapy, even in an extended treatment window, reduced the acute lesion volume and cerebral atrophy at 28 days post-stroke. NSC EV-treated stroke mice performed better in various behavioral tasks related to motor function, muscular strength, depression, and learning/memory. Taken together, our data in murine TE stroke strongly supports further development of NSC EV-based stroke therapy.

MRI assessments of infarct volume, atrophy, and brain swelling are pivotal predictors of clinical severity and prognosis and are critical readouts in assessing the efficacy of stroke therapies [42, 43]. NSC EVs administered both within and outside the tPA therapeutic window resulted in a significant decrease in infarct volume in our murine model. In addition, MRI results suggest NSC EVs also resulted in a significant reduction in tissue loss 28 days post-TE-MCAO in aged mice. These findings directly support recent reports in which MSC EVs were found to promote tissue preservation and neurovascular remodeling through proposed paracrine effectors [15, 44, 45].

NSC EVs may promote increases in vascular density and angiogenic processes by mediating specific gene regulation. For example, emerging data suggests downregulation of miR-15a in cerebral vessels in a murine model of ischemic stroke promotes angiogenesis in the peri-infarct region by increasing FGF-2 and VEGF levels [46, 47]. Many MSC EV-related studies have observed improvements in functional recovery, neurogenesis, and angiogenesis in rodent models of ischemic stroke [14, 15, 48, 49]. However, these studies have yet to report a significant difference in acute infarct volume as we have shown here. These results suggest that NSC EVs maybe therapeutically more potent than their MSC EV counterparts. While the exact molecular mechanism(s) responsible for these effects are currently unknown, it is possible that they are mediated by tetraspanin superfamily proteins. We routinely detect tetraspanins CD63 and CD81 in NSC EVs. Tetraspanins affect cell adhesion, motility, proliferation, and coagulation [50], which we believe may improve stroke outcomes.

It is imperative for the success of translational research to also incorporate behavioral tests that are sensitive to both the area of brain damage and the interventions that are being applied [51]. Neurological deficit scores and adhesive tape removal times revealed significant improvements in NSC EV-treated mice 2 and 4 days post-TE-MCAO, respectively. Furthermore, NSC EVs promoted significant improvements in balance beam walking, the number of footfalls, hanging wire, and tail suspension performance 14 days post-TE-MCAO in aged rodents. In comparison, similar studies evaluating rodent MSC EVs also reported significant behavioral improvements in comparatively young animals, in the absence of changes in infarct volume [14, 34, 52]. However, how rodent MSC EVs evaluated in young adult animals translate to the therapeutic potential of human MSC EVs and how those compare to NSC EVs are frequently not addressed—leaving plausible gaps in our knowledge of how these resources inform further development in preclinical programs for evaluation of EVs for therapeutic use in humans.

In addition to sensorimotor tests, we also evaluated NSC EV effects on declarative memory. Fourteen days post-TE-MCAO, our NSC EVs induced a significant improvement not only in NOR but also in associated NO discrimination performance. This suggests NSC EVs may also support the conservation of key brain regions associated with declarative memory and discrimination, like the dorsolateral prefrontal cortex and the medial temporal lobe [53, 54]. Advanced imaging and pharmacological inactivation studies in multiple animal models have also confirmed this theory by providing evidence that the prefrontal cortex plays a critical role during remote memory recall by regulating the hippocampus [55]. Stroke-induced injury to white matter tracts (including connections to the frontal and temporal cortices) has been linked to lasting deficits in episodic and declarative memory in both rodent models, as well as human patients [55–58].

This study uniquely encompassed a direct comparison of human MSC and NSC EVs while abiding by STEP and STAIR committee recommendations for developing stroke therapeutics. The extensive testing of NSC EVs has shown impressive biological relevance in the TE-MCAO model of ischemic stroke. By not only decreasing hemispheric swelling, atrophy, and infarct volume but also improving functional performance in vivo, NSC EVs possess potent and translatable therapeutic potential that with further testing may change the current therapeutic paradigm of ischemic stroke. Further testing in large animal models of stroke, as well as studies evaluating the use in conjunction with tPA and endovascular therapies, will further inform the therapeutic development potential of NSC EVs.

## Supplementary Materials

ESM 1 Three dimensional projection of in vitro uptake of NSC EV by differentiated neural cells (AVI 25643 kb)

Fig. S1 Cerebral blood flow analysis and survival curves (DOCX 135 kb)
