# Supplementary material for: Human Neural Stem Cell Extracellular Vesicles Improve Tissue and Functional Recovery in the Murine Thromboembolic Stroke Model
Source: Transl Stroke Res. 2017 Dec 28;9(5):530–9. doi: 10.1007/s12975-017-0599-2 (PMC6132936; doi:10.1007/s12975-017-0599-2)
Supplement: Supplementary file 2 — Cerebral blood flow analysis and survival curves (DOCX 135 kb) [file 12975_2017_599_MOESM2_ESM.docx]

**Supplemental Materials**

**Materials and Methods**

**Cell Culture, EV enrichment, and characterization**

H9 cells were differentiated into NSCs or MSCs using standard operating procedures previously published (*35-37*). Medium was harvested off of NSC cultures when cells reached ~80% confluence. MSC cultures were washed twice with PBS and cultured in serum-free medium for 24 hours prior to medium harvest for EV enrichment. Media was filtered through a 0.22 µm filter and then further enriched by ultrafiltration using a 100 kDa regenerated cellulose Amicon or Centricon ultra-centrifugal filter units or the Amicon stirred cell system, and washed twice with PBS**.** Enriched EVs were stored in single use aliquots, 200 µl for mice (2.7 x 10^11^ ± 10% vesicles/kg). Labeled EVs were incubated with 10 uM DiI for 30 min. before washes. DiI labeled EVs were applied to differentiated neural cells or MSCs and visualized by super resolution confocal microscopy.

**Electron microscopy**

For electron microscopy vesicle preparations were fixed in 2% paraformaldehyde for 15 minutes. EVs were transferred to Formvar-coated grids for 20 minutes, and then washed by transferring to drops of PBS. Grids were transferred onto drops of 1 % glutaraldehyde for 5 minutes, and then moved over several drops of water to remove residual glutaraldehyde before transferring to uranyl-oxylate. Grids were imaged by electron microscopy at 80 kV.

For NP sectioning, cells were collected and centrifuged at 1,000 rpm for 4 minutes. Cells were fixed in 2.5% glutaraldehyde in 0.1 M cacodylate-HCl (pH 7.2) buffer for 2 hours, then resuspended in 2% agarose, and post-fixed in 1% osmium tetraoxide for 1 hr. Cells were ethanol dehydrated sequentially (30%, 50%, 75%, 95% x 2, 100% x 2) for 15 minutes per step, and then transferred into propylene oxide (PO) for two 15 min. exchanges. The sample was transferred into PO/Epon Araldite mixture overnight, followed by two 60 min Epon/ Araldite exchanges, and embedded at 70°C for 12 hrs. Embedded cells were sectioned on an ultramicrotome, and viewed on a JEOL JEM1011 (JEOL USA, Inc.; Peabody, MA).

**Animal Study Design**

The overarching aim of these studies was to determine if either NSC or MSC EV treatment possessed biologically relevant therapeutic potential in a pre-clinical mouse TE-MCAO model of ischemic stroke. Endpoints were selected in order to evaluate both tissue and functional level changes in response to treatment. We used a split plot experimental design, where all treatment groups were conducted within a day to control and reduce variation, since day to day variation often occurs in animal studies. The first objective was to compare therapeutic efficacy of NSC EV and MSC EVs. In the murine studies, sample sizes were determined based on power calculations utilizing confidence intervals based on the investigator’s routine use of the TE-MCAO model and 12±1 month old C57BL/6 male mice (N=12 animals/group). From our previous experience with adult male mice in an eMCAO model, we anticipated stroke injury size to be 50% +/- 10% in the TE-MCAO+Veh group. Individual treatment was hypothesized to reduce the infarct size by 20% to 40% +/-10%. A sample size of 7 in each group would provide 88% power at 0.05 to detect the main effects due. Also based on our experience, mortality up to 30% was anticipated for some groups over the survival period of 48 hours. Therefore, 12 (2 extra/group) mice were randomized to each group to achieve a power of 80%.

Investigators were blinded randomized animals into groups within a day and the EVs were delivered to the laboratory in single use aliquots, labeled A-C (PBS control, MSC EV, or NSC EV). All experiments were completed and resulting data delivered prior to unblinding. Following initial studies in 12 month old mice, a more thorough evaluation of NSC EVs was warranted in an aged mouse model of TE-MCAO, 18±1 month old C57BL/6 male mice. Acute evaluation of treatment efficacy was completed using MRI as well as an expanded battery of behavioral tests (described in detail in the supplementary methods section). Furthermore, ex vivo MRI analysis was employed to assess brain atrophy. For this study, the experimental group was increased to 24 animals/group, and a sham group was added consisting of 10 animals. For subjective assessment of biodistribution by SPECT, 3 animals received indium labeled NSC EVs, and 3 animals received free indium (N=6 total for biodistribution). All endpoints and functional measurements were prospectively planned, and exclusion occurred only due to animals not surviving to the endpoint. No outliers were removed from the data.

**NSC EV vs. MSC EV Evaluation in a Murine Embolic Stroke Model**

Middle-aged C57BL/6 male mice (9 – 11 months) were acclimated for 1 week, housed with ad libitum access to food and water and under routing 12 hour light/dark cycle, and all procedures were approved by the Institutional Animal Care and Use Committee of Augusta University. Mice were numbered, and randomized for therapy into blinded treatment groups following induction of embolic stroke. All animals were pre-trained for adhesive tape test (ATT) for 3 days prior to the stroke surgery (3 trials/ day). TE-MCAO was induced by standard procedure [23, 24]. Briefly, mice were sedated with buprenorphine (0.05 mg/ kg SC) and plain of anesthesia was maintained with 1.5 – 2.0% isoflurane. Body temperature was maintained at 37 ºC by a thermo-regulated surgery pad. Midline incision on the ventral side of the neck exposed the right common carotid artery (CCA), external carotid artery (ECA), and the internal carotid artery (ICA). A temporary atraumatic clip was placed on the CCA to prevent loss of blood during catheter insertion. A modified PE-10 catheter containing a single fibrin rich clot (9 ±0.5mm length) was introduced into the ECA and advanced into the ICA. The clot was gently injected with 100 µL of PBS, the catheter was removed immediately after embolization, and the arterial wound was secured to prevent blood loss. Induction of stroke was confirmed using on-site portable single point cortical laser Doppler flowmetry (PeriMed Inc.). Finally, the temporary clip was removed and the blood flow in the CCA was reinstated. The site of surgery was closed using #6 sterile monofilament nylon suture, and buprenorphine (0.05 mg/ kg s.c.) was again injected. Mice were transferred to clean recovery cages and animal temperature was maintained. Conscious mice were transferred to clean regular cages with free access to food and water. NAPA gel and lactated ringers solution (LRS) were provided as needed for dehydration. Single use aliquots of EV preparations (either PBS, MSC EV, and NSC EV) were thawed and injected intravenously at 2, 14, and 38 hours post-stroke. Relative cerebral blood flow (CBF) was measured at 6 and 48 hours post stroke. Neurological deficit score (NDS) was assessed at 48 hours post-stroke; the ATT was evaluated 96 hours post-stroke just prior to euthanasia. Mice were deeply anesthetized with 5% isofluorane. Blood was collected via direct cardiac puncture and brains were perfused with 25 mls of cold 0.01 M phosphate-buffered saline (PBS), and immediately transferred to a metallic mouse brain matrix. Coronal sections (2 mm x 4 sections) were transferred into pre-warmed 5% TTC in PBS (Sigma) for 20 - 30 minutes at 37 °C, followed by fixation with 10% formalin. Corrected infarct volume was estimated using gray scale images and Scion Image software.

**EV Biodistribution study in rodents**

1.5-2 mCi of Indium-111-oxine in PBS was added to 200 µl EVs (~2.7 x 10^11^ vesicles/kg) and incubated at 37°C for 20 minutes. Free indium was removed by three repeated PBS washes through an Amicon ultrafiltration device. Collected EVs were diluted to 200 µCi of radioactivity per dose, and injected intravenously into the mouse-tail vein, either 1 hour or 24 hours post-stroke. Control animals received injection of free indium-111-oxine. Whole body and head single photon emission spectroscopy (SPECT) images were acquired by Mediso’s nanoScan microSPECT/CT system at 1 and 24 hours after injection, and projection images were reconstructed according to maximum intensity to determine radioactivity in the brain and throughout the body.

**Laser Speckle Contrast Imaging**

Mice were anesthetized using isoflurane 6 hours post-TE-MCAO, while body temperature was maintained at 37 ±0.5 °C. The skull was shaved and a midline skin incision was made to expose the middle cerebral region. Perfusion images were acquired using PeriCam high resolution Laser Speckle Contrast Imager (LSCI; PSI system, Perimed) with a 70 mW built-in laser diode for illumination and 1388 x 1038 pixels CCD camera installed 10 cm above the skull (speed 19 Hz, and exposure time 6 mSec, 1.3 x 1.3 cm). Acquired video and images were analyzed for dynamic changes in CBF. Overall perfusion of the ischemic region was compared to an equally sized region of interest from the uninjured contralateral hemisphere to estimate relative CBF. The skin wound was closed using tissue glue. At 48 hours post-stroke, the skin incision was reopened, cleaned, and the middle cerebral artery region was exposed to repeat the LSCI procedure as previously described.

**Neurologic Deficit Score**

Neurological deficits in mice were assessed by investigators blinded to the therapeutic group at 48 hours post-stroke on a 5-point scale with the highest number indicating the worst outcomes and lower number indicating better neurological outcomes according to the following criteria: 0, no deficit (normal mice); 1, forelimb flexion deficit on contralateral side; 2, flexion deficit along with decreased resistance to lateral push and torso turning to the ipsilateral side when held by tail; 3, All deficits as in Score 2, including very significant circling to the affected side during the move inside the cage, and reduced capability to bear weight on the affected side; 4, All deficits as above but rarely willing to move spontaneously, and prefer to stay in rest; 5, considered terminal and euthanized in accordance with animal care requirements.

**Adhesive Tape Test**

Adhesive tape test (ATT) was used as a test of somatosensory motor function, and was performed at 96 hours post-stroke immediately prior to euthanasia. Naïve mice were acclimatized to the procedure of the test for 3 days prior to surgery by placing them in a transparent acrylic box (15 cm x 25 cm). Two pieces of adhesive tape (0.3 cm x 0.4 cm) were used as bilateral tactile stimuli after they were attached at the distal-radial region on each forelimb such that it covered the hairless part (3 pads, thenar and hypothenar). Within 180 seconds, the tape removal time was recorded as the sensorimotor function. If a mouse failed to remove the tape within 180 seconds, it was given a score of 180 seconds. Therefore, a shorter time score indicates a better outcome while longer time indicates an outcome with higher deficit.

**Blood sample flow cytometry (Th17, Treg, M2)**

Prior to euthanasia blood was collected and purified cells were subjected to fluorescence activated cell sorting to identify populations of immune cells present systemically including T-helper (CD4+/FOX3P+) populations, regulatory T-cells (CD4+/IL17+), and M2 macrophage (IL10+/CD206+) populations.

**NSC EV Evaluation in an aged TE-MCAO mouse model**

C57BL/6 male mice aged 18+/- 2 months old were subjected to TE-MCAO as described with minor adjustments, including a reduced clot size of 7+/- .5 mm. Sham-operated mice were subjected to similar surgical procedure to infuse 1X sterile PBS without a clot.

**MRI Acquisition and Analysis**

All MRI experiments were conducted using a 7 Tesla horizontal magnet with a clear bore of 20 cm in diameter interfaced to a Bruker Advance console. Animals underwent fast spin echo (RARE factor = 8) with effective echo time of 47 ms to create corresponding T2 weighted images; arterial spin labeling (ASL) imaging acquired through an FAIR RARE sequence, together with the associated T1 map generated from a Look-Locker pulse sequence, to determine the CBF; *ex vivo* diffusion tensor imagining (DTI) through a pulse-gradient spin echo sequence generated diffusion parameters, including apparent diffusion coefficient (ADC), tensor trace, and fractional anisotropy (FA), in the fixed mouse brains.

Image post-processing for edema and CBF were performed using ImageJ coupled with in-house designed ImageJ macro scripts. T2 weighted images were used to determine the volume of cerebral edema by drawing an irregular region of interest (ROI) by encircling regions exhibiting edema in each image. Summation of the ROIs in all the impacted slices were multiplied by slice thickness. CBF was calculated based on the difference between two inversion recovery (slice-selected and non-slice-selected, respectively) pulsed images and the function of associated T1 values. DTI was analyzed using the vendor-supplied software Paravsion 5.1 (Bruker Inc.), from which the associated diffusion weighted, FA and ADC images were generated. Cerebral atrophy in the injured ipsilateral side was calculated as the percent volume loss compared to the uninjured contralateral side.

**Murine Behavior Assessments**

*Beam Walk Test*

The beam apparatus consisted of a graduated beam, placed and fixed 20-cm above the tabletop on two poles (6-mm flat width x 125-cm long). A black box is placed and fixed at the end of narrow beam as the finish point containing nesting (bedding) material from the home cage to attract the mouse. A lamp (with 60-watt light bulb) is used to shine light above the start point, serving as an aversive stimulus. Mice were pre-trained (3 trials on 3 days) on the balance beam and evaluated 14 days post-TE-MCAO. Time to traverse the 100-cm distance and the number of foot slips were quantified, and presented as mean values ± standard error of the mean.

*Hanging Wire*

A 55-cm long 1-mm diameter single metal cord was stretched firmly between two metal stands, 50-cm above a standard mouse cage filled with soft paper bedding material. Mice were trained for 3 trials x 3 consecutive days before the actual test, or until they are trained to grab it with two forelimbs. For the actual test, the latency to lose the grip on the wire falling into the cage was recorded for 3 trials x 5-min interval between trials for each mouse.

*Tail Suspension Test*

Mice were habituated for 3 days with single 5-min trial per day. At 4 weeks post-TE-MCAO mice were firmly suspended 0.75-cm away from the origin of their tails, and elevated 70-cm above the floor. Behavior was recorded as the immobility time during a single trial of 5-min in an isolated room dedicated for behavioral testing. Data are presented as the absolute immobility time (in seconds) or as the percent immobility.

*Novel Object Recognition*

Behavioral assessment by NOR was performed 4 weeks post-TE-MCAO. Mice were habituated in an activity box and familiarized with two objects placed at a set distance apart. On the day of the trial time spent (*T_f_*) with the familiar object was recorded. After the familiar object trial, the mouse was then removed from the environment and one of the two previously used (familiar) objects was replaced with a novel object. The time spent (*T_n_*) with the novel object was then recorded in one 5 minute trial. The capability of the mouse to discriminate between a familiar vs. novel object was determined as the discrimination index, *DI = (T_n_-T_f_)/(T_n_+T_f_)*. A lower DI-value reflects poor cognitive function, while a higher DI-values demonstrates a better learning and memory function.


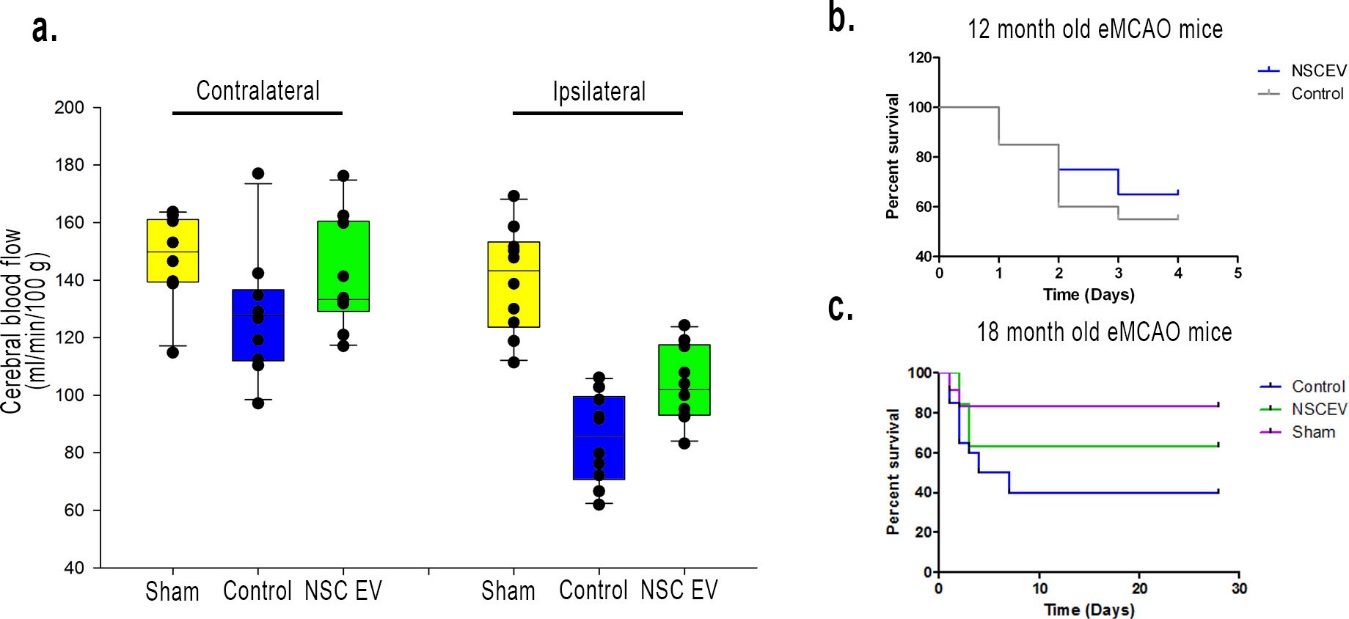


Fig. S1. Cerebral blood flow is not different between NSC EV and control groups at 48 hours post-stroke (a). Kaplan-Meier curves depict rodent survival through the duration of their study (b). In the 12 month old rodent model, there was no difference in overall survival between the control and NSCEV group with the log rank test (P=0.528). In the aged rodent study, control mice had lower survival than sham mice with the log rank test (P=0.029), while the NSCEV group did not (P=0.319) (c).  There was no statistical difference in survival between the control and NSCEV group with the log rank test (P=0.1409).
